# Rising Incidence of Legionnaires’ Disease and Associated Epidemiologic Patterns, United States, 1992–2018

**DOI:** 10.3201/eid2803.211435

**Published:** 2022-03

**Authors:** Albert E. Barskey, Gordana Derado, Chris Edens

**Affiliations:** Centers for Disease Control and Prevention, Atlanta, Georgia, USA

**Keywords:** Legionnaires’ disease, surveillance, epidemiology, United States, bacteria, *Legionella*, legionellosis, pneumonia, respiratory infections

## Abstract

Reported Legionnaires’ disease (LD) cases began increasing in the United States in 2003 after relatively stable numbers for >10 years; reasons for the rise are unclear. We compared epidemiologic patterns associated with cases reported to the Centers for Disease Control and Prevention before and during the rise. The age-standardized average incidence was 0.48 cases/100,000 population during 1992–2002 compared with 2.71 cases/100,000 in 2018. Reported LD incidence increased in nearly every demographic, but increases tended to be larger in demographic groups with higher incidence. During both periods, the largest number of cases occurred among White persons, but the highest incidence was in Black or African American persons. Incidence and increases in incidence were generally largest in the East North Central, Middle Atlantic, and New England divisions. Seasonality was more pronounced during 2003–2018, especially in the Northeast and Midwest. Rising incidence was most notably associated with increasing racial disparities, geographic focus, and seasonality.

## Abstract 2

Rising incidence was associated with increasing racial disparities, geographic focus, and seasonality.

Legionnaires’ disease (LD) is a severe pneumonia caused by *Legionella* spp. bacteria. Approximately 95% of patients require hospitalization, and 10% die (*1*). Risk factors include older age (>50 years), smoking, a weakened immune system, and chronic lung conditions (*2*). Pontiac fever (a self-limited, influenza-like illness) and extrapulmonary legionellosis (*Legionella* infection with a primary focus outside the lungs) are other less common legionellosis syndromes (*1*).

*Legionella* is found in most freshwater environments in low numbers. The bacteria can proliferate in built environments, particularly when the water is warm (25°C–45°C), stagnant, and lacking residual disinfectant. Some devices, such as cooling towers, hot tubs, showers, and decorative fountains, can aerosolize water and have frequently been associated with LD outbreaks (*3*). LD can be acquired when aerosolized water containing *Legionella* bacteria is inhaled. A properly designed and implemented water management program (WMP) can reduce the risk for *Legionella* growth and transmission in buildings with complex water systems (*3*–*5*). WMPs were first recommended in 2015 (*4*).

*L. pneumophila* was discovered in 1977 and recognized as the etiologic agent in an outbreak of severe pneumonia the previous year (*6*,*7*). LD cases reported to the Centers for Disease Control and Prevention (CDC) steadily increased from 235 in 1976 to 1,370 in 1990 (*8*). Reported cases in the United States remained relatively stable during 1990–2002 but began increasing steadily in 2003 (*9*–*11*); however, the reasons are unclear. To explore factors that might have contributed to the increase, we compared epidemiologic patterns associated with the baseline years before the increase (1992–2002) and those associated with the years of increase (2003–2018).

## Methods

US jurisdictions (the 50 states plus New York, NY, and Washington, DC) report cases of legionellosis (referred to as LD) (*1*) to CDC through the National Notifiable Diseases Surveillance System (NNDSS). We included data from 1992 (the earliest year of electronically available data) through 2018. Although 2019 data are available, completeness of the data reported by more than one third of US jurisdictions is uncertain because of the coronavirus disease pandemic (*12*). LD was not reportable in Connecticut during 1992–1996 or in Oregon or West Virginia during 1992–2002; we excluded cases and populations from these jurisdictions and years from analyses. 

During the study period, the LD case definition changed (in 1997 and 2006); we included cases meeting the case classification criteria for reportable conditions in use at the time the cases occurred (*13*–*15*). All 3 case definitions defined a confirmed case of LD as a clinically compatible illness with isolation of any *Legionella* organism from respiratory secretions, lung tissue, pleural fluid, or other normally sterile fluid; detection of *L. pneumophila* serogroup 1 antigen in urine using validated reagents; or a >4-fold rise in specific serum antibody titer to *L. pneumophila* serogroup 1 using validated reagents (*13*–*15*). The 1996 case definition included the detection of *L. pneumophila* serogroup 1 in respiratory secretions, lung tissue, or pleural fluid by direct fluorescent antibody testing, and it required the >4-fold rise in antibody titer to reach >128. The 1990 case definition included probable cases, defined as a clinically compatible illness with demonstration of a reciprocal antibody titer >256 from a single convalescent-phase serum specimen.

Available patient data included age, sex, race, ethnicity, jurisdiction of residence, and date of earliest reported event in case history (event date). We did not analyze ethnicity because data were missing for 30.4% of cases. Cases were associated with the event date rather than the date reported to the health department or CDC. Event dates consisted of onset date (78%), diagnosis date (9%), laboratory result date (6%), date first reported to any public health authority (3%), and date reported to the state health department or CDC (3%); 1% of cases were missing date type. 

**Figure 1:**
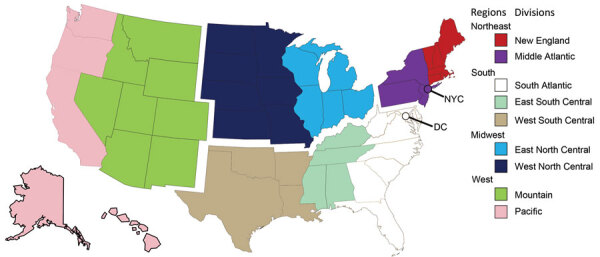
US Census Bureau regions and divisions. Regions: Northeast: Connecticut, Maine, Massachusetts, New Hampshire, New Jersey, New York City, New York State, Pennsylvania, Rhode Island, Vermont; Midwest: Illinois, Indiana, Iowa, Kansas, Michigan, Minnesota, Missouri, Nebraska, North Dakota, Ohio, South Dakota, Wisconsin; South: Alabama, Arkansas, Delaware, District of Columbia, Florida, Georgia, Kentucky, Louisiana, Maryland, Mississippi, North Carolina, Oklahoma, South Carolina, Tennessee, Texas, Virginia, West Virginia; West: Alaska, Arizona, California, Colorado, Hawaii, Idaho, Montana, Nevada, New Mexico, Oregon, Utah, Washington, Wyoming. Divisions: New England: Connecticut, Maine, Massachusetts, New Hampshire, Rhode Island, Vermont; Middle Atlantic: New Jersey, New York City, New York State, Pennsylvania; East North Central: Illinois, Indiana, Michigan, Ohio, Wisconsin; West North Central: Iowa, Kansas, Minnesota, Missouri, Nebraska, North Dakota, South Dakota; South Atlantic: Delaware, District of Columbia, Florida, Georgia, Maryland, North Carolina, South Carolina, Virginia, West Virginia; East South Central: Alabama, Kentucky, Mississippi, Tennessee; West South Central: Arkansas, Louisiana, Oklahoma, Texas; Mountain: Arizona, Colorado, Idaho, Montana, Nevada, New Mexico, Utah, Wyoming; Pacific: Alaska, California, Hawaii, Oregon, Washington.

Jurisdictions were grouped by US Census Bureau regions and divisions (Figure 1). To quantify seasonality, we calculated the annual maximum-to-minimum monthly case ratio by dividing the maximum number of monthly cases by the minimum number of monthly cases within a calendar year. For most analyses, we aggregated data within 2 time periods (baseline years [1992–2002] and increase years [2003–2018]) and then compared them. We selected 2002, the last year before annual cases numbered >2,000, as a breakpoint for our analyses to aid in comparisons with previously published work (*9*–*11*). To quantify the magnitude of increase, we compared the age-standardized incidence in 2018 with the age-standardized average incidence for 1992–2002 (Appendix). We used bridged-race postcensal population estimates to calculate incidence (*16*). Incidence was age-standardized by using the 2005 US standard population as the reference population.

We performed statistical analyses by using SAS (version 9.4; SAS Institute, https://www.sas.com). We performed joinpoint regression analysis, also known as change point regression or segmented regression (Joinpoint software version 4.8.0.1, https://surveillance.cancer.gov/joinpoint) on the age-standardized incidence and mean and median age over time to identify the optimal year when population parameters changed (Appendix).

## Results

**Figure 2:**
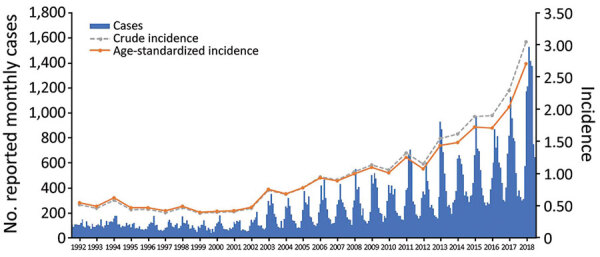
Reported cases of Legionnaires’ disease by month and incidence (cases/100,000 population) by year, United States,1992–2018. Monthly cases reported to the Centers for Disease Control and Prevention through the National Notifiable Diseases Surveillance System and the crude and age-standardized annual incidence for 1992–2018 are shown.

During 1992–2002, an average of 1,221 (range 1,060–1,547) LD cases were reported annually; during 2003–2018, an average of 4,369 (range 2,082–9,999) cases were reported annually. Crude and age-standardized incidence increased from 0.52 and 0.55 cases/100,000 population in 1992 to 3.06 and 2.71 cases/100,000 population in 2018 (Figure 2). Over the study period, joinpoint analysis selected a model with 1 change point in the trend in age-standardized incidence as the best model (over models with zero or 2 change points). Although joinpoint analysis identified the single optimal change point in the trend in age-standardized incidence (p<0.05) as 1999 (95% CI 1996–2002), we retained 2002 as the breakpoint in our analyses to aid in comparisons with previous studies. In addition, the largest relative increase (26%) in a 3-year moving average of age-standardized incidence over the study period occurred in 2003. From 1992 to 2002, no indication of a trend in age-standardized incidence was seen (−0.2%, 95% CI −5.1% to 5.0%); from 2002 to 2018, the average annual increase in age-standardized incidence was 9.3% (95% CI 8.1%–10.4%), of which the largest increase occurred during 2016–2018.

### Age

**Figure 3:**
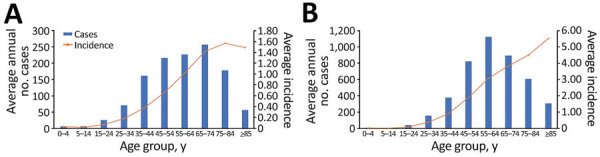
Average annual number of cases of Legionnaires’ disease and average incidence (cases/100,000 population), by age group, United States, 1992–2018. A) Reported average number of annual cases and average incidence by age group for 1992–2002. B) Reported average number of annual cases and average incidence by age group for 2003–2018.

Age data were available for 82,649 (99.2%) of the 83,334 cases in the study period. During the baseline years, the largest number of average annual cases (257) was reported in the 65–74-year age group; the average number of cases in the 2 older age groups (75–84 and >85 years) was lower than the 2 younger age groups (45–54 and 55–64 years) (Figure 3, panel A). Average age-specific incidence generally increased with age, rising from <0.1 cases/100,000 population in children and young adults (0–24 years) to peak in the 75–84-year age group (1.57 cases/100,000 population) (Appendix Table). During the increase years, the largest number of average annual cases (1,122) was reported in the 55–64-year age group, and the distribution was more symmetric around this peak (Figure 3, panel B) than around the peak for the baseline years. Except for the 0–14-year group, in which incidence remained low (<0.1 cases/100,000 population), average age-specific incidence increased with age through the >85 years category (5.52 cases/100,000 population).

**Figure 4:**
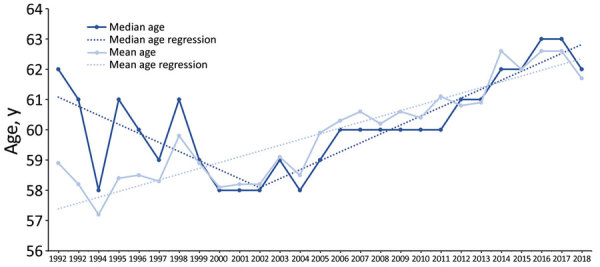
Trends in median and mean age of Legionnaires’ disease patients by year, United States, 1992–2018.

Joinpoint analysis identified 2002 as the change point in the trend of median patient age (Figure 4). Median patient age decreased from 62 years in 1992 to 58 years in 2002, then increased to 62 years in 2018. We identified a model with no change points as the best model for the trend in mean patient age over the study period; mean age increased from 58.9 years to 61.7 years.

### Sex

During 1992–2002, men accounted for 59.8% of the 13,137 cases for whom sex were reported, compared with 62.8% of 69,226 cases during 2003–2018. The age-standardized average incidence in men was 0.63/100,000 men and in women was 0.35/100,000 women during 1992–2002 (Appendix Table). During 2003–2018, the age-standardized average incidence increased to 1.80/100,000 in men and 0.91/100,000 in women.

### Race

**Figure 5:**
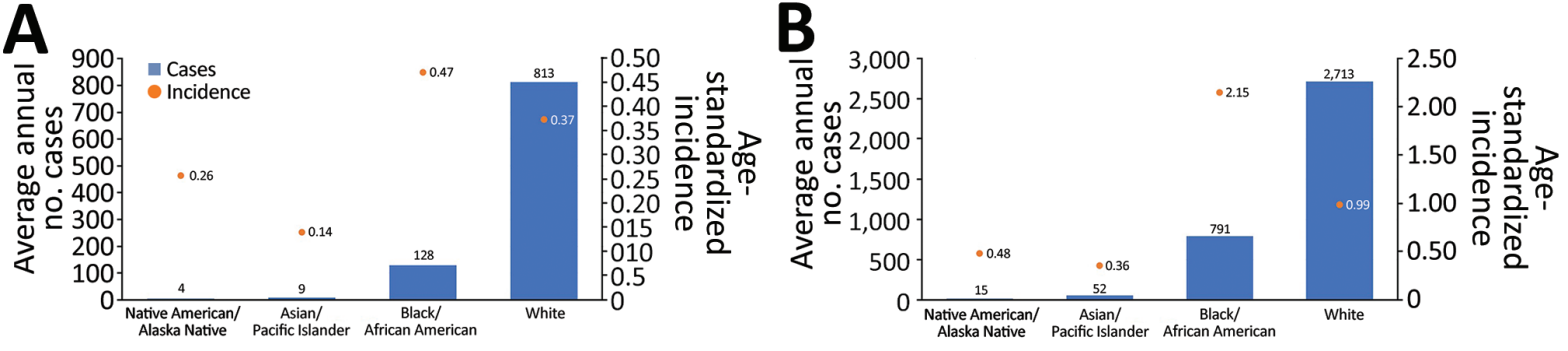
Average annual number of cases of Legionnaires’ disease and age-standardized average incidence (cases/100,000 population) by race, United States, 1992–2018. A) Reported average number of annual cases and age-standardized average incidence by race for 1992–2002. B) Reported average number of annual cases and age-standardized average incidence by race for 2003–2018.

Race or age was missing for 16.5% of cases; thus, race-specific case counts and incidences might be slightly higher than measured in this study. During the baseline years, >6 times the number of average annual cases were reported among White persons (813) than Black or African American persons (128), but the age-standardized average incidence was >25% higher among Black or African American persons (0.47/100,000 population) than White persons (0.37/100,000 population) (Figure 5, panel A; Appendix Table). This pattern continued, and racial disparities were more pronounced during the years of increase, when the age-standardized average incidence was twice as high among Black or African American persons (2.15/100,000 population) than among White persons (0.99/100,000 population) (Figure 5, panel B).

### Geographic Distribution

**Figure 6:**
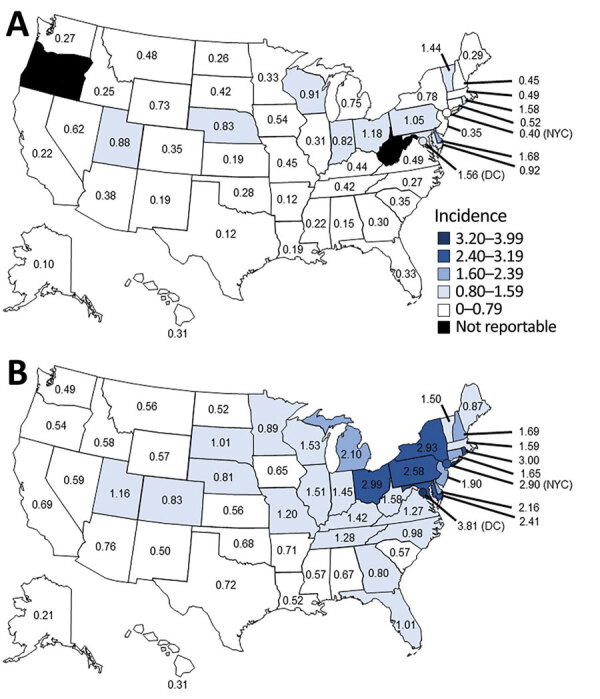
Age-standardized average incidence (cases/100,000 population) of Legionnaires’ disease by jurisdiction, United States, 1992–2018. A) Age-standardized average incidence by jurisdiction, 1992–2002. Legionnaires’ disease was not reportable in Connecticut during 1992–1996 or in Oregon or West Virginia during 1992–2002. B) Age-standardized average incidence by jurisdiction, 2003–2018.

During both the baseline years and the years of increase, the age-standardized average incidence was higher in the Northeast (0.68/100,000 population in baseline years; 2.34/100,000 population in years of increase) and Midwest (0.67; 1.67) regions than in the South (0.33; 1.01) and West (0.29; 0.66) regions (Appendix Table). Similarly, the contiguous East North Central (0.77; 2.01), Middle Atlantic (0.71; 2.59), and New England (0.61; 1.64) divisions had the highest age-standardized average incidence during the baseline years and the years of increase. Among the 20 jurisdictions with the highest age-standardized average incidence during 1992–2002, a total of 10 were located within the East North Central, Middle Atlantic, or New England divisions, and 3 others bordered these divisions (Figure 6, panel A). During 2003–2018, 14/20 jurisdictions with the highest age-standardized average incidence were located within these same 3 divisions, and 4 additional jurisdictions (of the 20) bordered these divisions (Figure 6, panel B).

### Seasonality

Most LD cases occurred during summer or fall months, and this pattern became more extreme after the baseline years (Figure 2). During 1992–2002, an average of 57.8% of annual cases occurred during June–November, increasing to 68.9% during 2003–2018. The average annual maximum-to-minimum monthly cases ratio rose from 2.59 during the baseline years to 4.31 during the years of increase.

**Figure 7:**
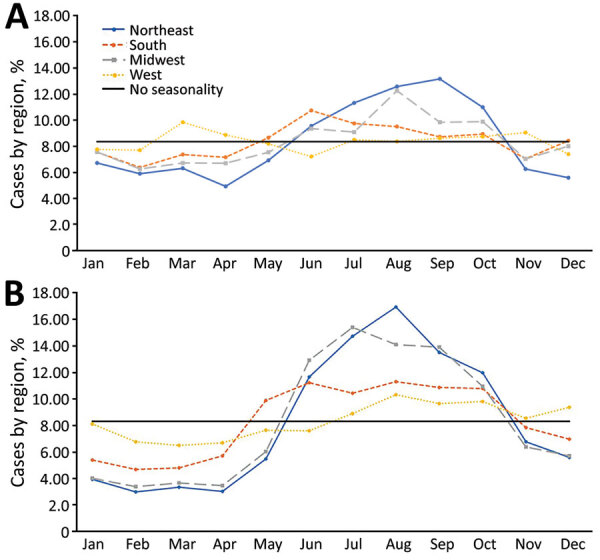
Seasonality of Legionnaires’ disease cases by Census Bureau region, United States, 1992–2018. A) Seasonality of cases by US Census Bureau region, 1992–2002. The monthly percentage of each region’s cases is shown. If no seasonality existed, approximately the same number of cases would be expected to occur each month (i.e., 1/12 [8.3%] of annual cases would occur each month). B) Seasonality of cases by US Census Bureau region, 2003–2018. The monthly percentage of each region’s cases is shown. If no seasonality existed, approximately the same number of cases would be expected to occur each month (i.e., 1/12 [8.3%] of annual cases would occur each month).

By geography, during the baseline years, moderate seasonality was observed in the Northeast region and less so in the Midwest and South regions (Figure 7, panel A). No seasonal pattern was discernible in the West. When cases increased during 2003–2018, seasonality became more prominent in all regions, particularly in the Northeast and Midwest (Figure 7, panel B). A less pronounced but identifiable seasonal pattern was also observed in the West. The LD season began first in the South and maintained a peak in this region from June through October. The LD season began later in the Midwest and Northeast, peaking in July in the Midwest and in August in the Northeast.

### Magnitude of Increase

Overall, age-standardized average incidence increased from 0.48/100,000 population during the baseline years (1992–2002) to 2.71/100,000 population in 2018 (incidence risk ratio [RR] 5.67, 95% CI 5.52–5.83) (Table). Relative changes in incidence in the 0–4-year and 5–14-year age groups were not statistically significant (RR 0.16, 95% CI 0.02–1.19 for 0–4 years; RR 0.48, 95% CI 0.15–1.54 for 5–14 years). Incidence increased >5-fold for all age groups above 34 years; the largest relative increases occurred in the >85-year (RR 6.50, 95% CI 5.82–7.27), 55–64-year (RR 6.39, 95% CI 6.05–6.75), and 45–54-year (RR 6.28, 95% CI 5.91–6.69) age groups. Age-standardized incidence increased slightly more in men (RR 5.86, 95% CI 5.67–6.05) than in women (RR 5.29, 95% CI 5.06–5.53). The age-standardized incidence increased from 0.47 to 5.21/100,000 population in Black or African American persons (RR 11.04, 95% CI 10.39–11.73) and from 0.37 to 1.99/100,000 population in White persons (RR 5.30, 95% CI 5.12–5.49).

**Table:** Magnitude of increase in age-standardized incidence of Legionnaires’ disease, cases/100,000 population, from 1992–2002 (average) through 2018, United States

Demographic	Age-standardized average incidence, 1992–2002	Age-standardized incidence, 2018	Absolute increase in age-standardized incidence	Age-standardized incidence risk ratio, 2018 to 1992–2002 baseline (95% CI)	Increase in age-standardized incidence, %
Age group, y, not standardized					
0–4	0.03	0.01	−0.03	0.16 (0.02–1.19)	−83.52
5–14	0.02	0.01	−0.01	0.48 (0.15–1.54)	−51.61
15–24	0.07	0.19	0.12	2.80 (2.19–3.57)	179.80
25–34	0.18	0.75	0.58	4.30 (3.79–4.88)	330.31
35–44	0.38	1.97	1.59	5.15 (4.74–5.59)	414.89
45–54	0.66	4.12	3.46	6.28 (5.91–6.69)	528.44
55–64	1.02	6.52	5.50	6.39 (6.05–6.75)	539.14
65–74	1.42	7.66	6.24	5.40 (5.11–5.70)	439.63
75–84	1.57	8.52	6.96	5.44 (5.07–5.84)	444.13
>85	1.49	9.69	8.20	6.50 (5.82–7.27)	550.35
Sex					
M	0.63	3.66	3.04	5.86 (5.67–6.05)	485.55
F	0.35	1.86	1.50	5.29 (5.06–5.53)	429.22
Race*					
Native American or Alaska Native	0.26	1.27	1.01	4.93 (3.51–6.93)	392.94
Asian or Pacific Islander	0.14	0.56	0.42	4.03 (3.19–5.10)	303.18
Black or African American	0.47	5.21	4.74	11.04 (10.39–11.73)	1003.95
White	0.37	1.99	1.61	5.30 (5.12–5.49)	430.15
Region					
Division					
Northeast	0.68	4.82	4.14	7.04 (6.70–7.40)	604.10
New England	0.61	4.33	3.72	7.10 (6.40–7.87)	610.04
Middle Atlantic	0.71	5.00	4.30	7.07 (6.69–7.48)	606.98
South	0.33	1.97	1.64	5.97 (5.67–6.29)	497.23
South Atlantic	0.44	2.29	1.85	5.24 (4.91–5.59)	423.54
East South Central	0.32	2.05	1.73	6.40 (5.63–7.27)	539.66
West South Central	0.15	1.36	1.21	9.15 (8.10–10.34)	815.03
Midwest	0.67	4.10	3.43	6.13 (5.85–6.42)	513.06
East North Central	0.77	5.01	4.24	6.48 (6.16–6.82)	548.02
West North Central	0.42	2.04	1.62	4.81 (4.29–5.40)	381.38
West	0.29	0.99	0.70	3.39 (3.11–3.68)	238.50
Mountain	0.43	1.07	0.64	2.47 (2.15–2.83)	146.55
Pacific	0.23	0.95	0.72	4.13 (3.71–4.59)	312.91
United States	0.48	2.71	2.23	5.67 (5.52–5.83)	467.30

*Ethnicity was not analyzed because data were missing for 30.4% of cases.

By region, the relative increase in age-standardized incidence was largest in the Northeast (RR 7.04, 95% CI 6.70–7.40), similar in the Midwest (RR 6.13, 95% CI 5.85–6.42) and South (RR 5.97, 95% CI 5.67–6.29), and smallest in the West (RR 3.39, 95% CI 3.11–3.68). By division, the largest relative increase in age-standardized incidence occurred in the West South Central division (RR 9.15, 95% CI 8.10–10.34). The next-largest relative increases were similar among the New England (RR 7.10, 95% CI 6.40–7.87), Middle Atlantic (RR 7.07, 95% CI 6.69–7.48), East North Central (RR 6.48, 95% CI 6.16–6.82), and East South Central (RR 6.40, 95% CI 5.63–7.27) divisions. The smallest relative increase in age-standardized incidence was in the Mountain division (RR 2.47, 95% CI 2.15–2.83). Although the largest relative increase in age-standardized incidence occurred in the West South Central division, the largest absolute increases occurred in the Middle Atlantic, East North Central, and New England divisions.

## Discussion

Reported incidence of LD in the United States has been rising since 2003, and the increase appears to be accelerating in recent years. Joinpoint analysis confirmed that a change in trend in age-standardized incidence occurred between 1996 and 2002, inclusively; no trend was identified before the change point, and an increasing trend was identified after. Although 1999 was indicated as the single optimal change point, and age-standardized incidence increased slightly every year after 1999 until 2004, the first substantial increase beyond what was likely the baseline range occurred in 2003. However, the rising incidence was not uniform and affected some demographic groups disproportionately. Increases tended to be larger in demographics with higher incidence. This rise was most strikingly associated with increases in racial disparities, geographic focus, and seasonality. We also noted changes in age and sex distributions.

The US population is aging (*16*–*18*); because older age is a risk factor for LD (*2*) and incidence increased with age, the aging population might contribute to the rising national incidence of LD. In this analysis, age-standardized incidence increased less than crude incidence. However, this difference was minor (12% in 2018), and relative increases in incidence from the baseline years to 2018 for all age groups older than 34 years were at least equal to the national average, suggesting that other factors played larger roles in the rising trend.

Although most LD cases occurred among White persons, Black or African American persons were disproportionately affected. Certain underlying conditions, including diabetes, end-stage renal disease, and some cancers, have been associated with an increased risk for LD (*2*), and these conditions are more common among Black or African American persons than White persons (*19*–*22*). Social determinants of health also likely contributed to disparities in incidence (*23*). Black or African American persons had the lowest median household income relative to other races (*24*), and areas of poverty were associated with a higher incidence of LD (*25*,*26*). Residence in areas with more vacant housing, more renter-occupied homes, more homes built before 1970, and lower education levels were also identified as risk factors for LD (*26*). Certain occupations (transportation, repair, protective services, cleaning services, and construction) were found to carry a higher risk for LD, but the associations with race and socioeconomic status were unclear (*25*). The relative increase in LD incidence from baseline years to 2018 was larger among Black or African American persons than any other demographic group, suggesting that the conditions leading to this disparity have been worsening.

Geographically, LD incidence was generally focused around an area extending from Ohio into New York state and Maryland and decreased with distance from this center. Although incidence rose nationwide, areas with higher incidence tended to have larger increases. These findings indicate that factors shared by geographic areas might have contributed to the rise in cases. Several studies found temperature, precipitation, and humidity to be associated with LD cases, although the mechanics are not completely understood (*27*–*31*). Aging infrastructure might also have played a role, because residing in areas with older homes has been identified as a risk factor for LD (*26*). Median population age varied by jurisdiction; the Northeast region had the highest median population age, followed by the Midwest, South, and West regions (*17*). However, standardizing age across jurisdictions for 2018 did not dramatically alter the jurisdiction-specific incidence from the crude incidence, suggesting that geographic variations in population age did not account for the higher incidence observed in the Middle Atlantic, East North Central, and New England divisions to a large extent.

LD exhibits a summer-through-early-fall seasonality, and this pattern became more pronounced as incidence increased, which could imply that the cyclical factors causing seasonal patterns are becoming more extreme. One likely candidate for a cyclical factor that could cause seasonal patterns in LD cases is weather. From 1990–2020, summer precipitation and the fall mean temperature have been increasing in high-incidence divisions (*32*). Our results and previous findings suggest that the peak of the LD season shifted from late summer to mid-summer, particularly in the Northeast and Midwest regions (*11*). Wetter summers might partly explain this shift, because precipitation and humidity have been associated with increased cases (*27*–*31*). Similarly, temperatures in the South reach *Legionella*-promoting temperatures, which also increase cooling tower use, earlier in the year than in the Northeast or Midwest, which might explain why the LD season begins first in the South (*33*). Furthermore, hurricane-produced rainfall increased during 1998–2016 (*34*), and hurricanes have been associated with elevated concentrations of *Legionella* bacteria in cooling towers and surface water (*35*,*36*). Travel is also a cyclical risk factor for LD but does not appear to influence seasonality; seasonal patterns for travel-associated cases were nearly identical to those for non–travel-associated cases during 2015–2016 (*37*). Furthermore, the percentage of travel-associated cases remained relatively stable over time (*37*).

**Figure 8:**
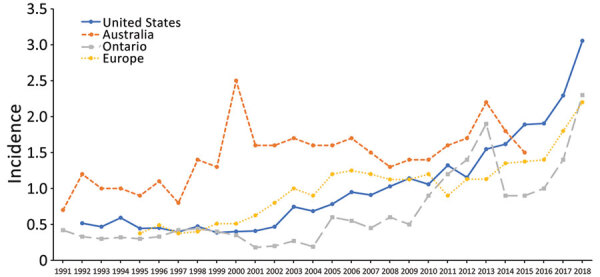
International crude incidence (cases/100,000 population) trends of Legionnaires’ disease, United States (National Notifiable Diseases Surveillance System), Europe (*39*), Ontario, Canada (*40*,*41*), and Australia (*42*), 1991–2018.

LD might occur worldwide because *Legionella* is a ubiquitous freshwater bacterium (*38*), but reporting and surveillance vary considerably. Patient demographics and a general rise in incidence were similar in the United States, Europe, Canada, and Australia, but the trajectory of the rising incidence trend was more similar in northern hemisphere locations than Australia (Figure 8) (*39*–*42*). This finding could suggest that factors common to northern regions, such as weather patterns, influenced the increase. In Ontario, Canada, just north of the high-incidence Middle Atlantic and East North Central divisions, LD incidence was generally highest in the southern part of the province, north of Lakes Erie and Ontario (*41*). Reasons for the worldwide increase in LD are unclear but might include an aging population, surveillance and reporting improvements, building infrastructure design and maintenance, and weather patterns (*39*,*40*).

The first limitation of our study is that, when evaluating rising incidence, separating the effect of improved surveillance from a true increase in infections is difficult. NNDSS is a passive surveillance system, and incomplete case-reporting is a concern with passive systems; however, a comparison with an active reporting system suggested that nearly all diagnosed LD cases were reported (*43*). LD might be underdiagnosed; studies estimate that 20,000 cases might occur annually (*2*,*44*). Because of the severe acute respiratory syndrome pandemic during 2002–2003 (*45*), practitioners might have increased the thoroughness of testing community-acquired pneumonia (CAP) patients to confirm an alternative diagnosis, thereby increasing the number of LD tests performed (*29*). Although this factor might explain the initial rise in reported LD cases in the United States, it does not explain the continued increase through 2018 or why increases did not occur simultaneously in other areas of the world, particularly Ontario, where severe acute respiratory syndrome cases occurred most outside of Asia (*45*). Although the case definition changed twice during our study period, the differences were small and unlikely to have substantially affected diagnosis or reporting. All case definitions included a positive urinary antigen test, isolation of *Legionella* spp., and a ≥4-fold rise in antibody titer to *L. pneumophila* serogroup 1 as options for confirming a case (*13*–*15*); most cases were confirmed by 1 of these methods (*1*,*46*). Before 2006, the direct fluorescent antibody test was also included, but its use in diagnosis had been declining since the mid-1990s (*46*). At the same time, the urinary antigen test came into widespread use and by 1998 was used in the diagnosis of >70% of reported cases (*46*). Therefore, changes in the case definition or available diagnostic tests are unlikely to account for the rising incidence after 2002.

Despite these limitations, our findings indicate several instructive points. Although professional guidelines recommend testing for *Legionella* in CAP patients associated with certain factors, such as an LD outbreak or recent travel, or in adults with severe CAP (*47*), clinicians might maintain a higher index of suspicion for LD in other CAP patients under certain circumstances because LD cases are rising nationwide and cannot be diagnosed on clinical features alone. Our results showed LD incidence was highest in older persons (particularly >55 years of age) and Black or African American persons, but these demographic groups also tended to have the highest rates of pneumonia-associated hospitalizations (*48*). Because LD incidence was highest in the East North Central, Middle Atlantic, and New England divisions, and pneumonia-associated hospitalization incidence was not similarly higher in these divisions (*48*), the likelihood that a CAP case is LD might be elevated in these locations. Similarly, more LD cases occurred during the summer and early fall, especially in the Northeast and Midwest, but most pneumonia-associated hospitalizations occurred during December–March (*48*); therefore, a larger percentage of CAP cases during the summer and early fall might be LD. Others have suggested increasing suspicion for LD in CAP patients during warm, humid, rainy weather (*27*).

In conclusion, LD incidence has risen steadily nationwide for >15 years, and the increase was associated with wider racial disparities, intensifying geographic focus, and more pronounced seasonality. The geographic focus and seasonality suggest that deeper investigations into the effects of weather may further elucidate the rising incidence of LD. Although WMPs are recommended for buildings with complex water systems and certain devices (*3*–*5*), uptake might be slow (*49*), and additional prevention methods could be useful. Outbreaks can cause substantial illness and deaths (*50*), but ≈64% of reported LD cases have no known potential exposure and generally lack an identified source (*1*). Improved investigations of sporadic cases and their sources may lead to novel prevention strategies and the identification of previously unrecognized outbreaks.

Appendix Additional information about the rising incidence of Legionnaires’ disease and associated epidemiologic patterns, United States, 1992–2018.
