# Supplementary material for: Rising Incidence of Legionnaires’ Disease and Associated Epidemiologic Patterns, United States, 1992–2018
Source: Emerg Infect Dis. 2022 Mar;28(3):527–38. doi: 10.3201/eid2803.211435 (PMC8888234; doi:10.3201/eid2803.211435)
Supplement: Appendix — Additional information about the rising incidence of Legionnaires’ disease and associated epidemiologic patterns, United States, 1992–2018. [file 21-1435-Techapp-s1.pdf]

Article DOI: https://doi.org/10.3201/eid2803.211435

Rising Incidence of Legionnaires' Disease and Associated Epidemiologic Patterns, United States, 1992–2018

Appendix

Statistical Methods

Incidence Risk Ratios and Corresponding 95% CI

To calculate the incidence risk ratios comparing the age-standardized incidence in 2018 with the average age-standardized incidence for the period 1992–2002, we first calculated the expected age-standardized case counts for year 2018 and the total expected age-standardized case counts for the period 1992–2002 by multiplying the age-standardized incidence by the population. We then calculated the incidence risk ratios and the corresponding 95% CIs by using asymptotic Wald confidence limits based on a log transformation of the relative risk. Calculations were performed by using the SAS procedure PROC FREQ.

Joinpoint Regression Analysis

To analyze the trend in Legionnaires' disease (LD) incidence and to determine the optimal change point(s) in the trend, we used a joinpoint (or segmented line) regression approach implemented by using the Joinpoint Regression Trend Analysis Software (*1*). Unknown change points in trend, or mean response, of observed characteristics *Y* on time or other predictors *X* are estimated from a set of observations (x_1,y_1), (x_2,y_2), …(x_n, y_n); $x_1 \leq x_2 \leq \ldots \leq x_n$. The change point (CP) is defined as the point in which an ordered sequence of data $(y_1, y_2, \ldots, y_n)$ changes its statistical properties; it occurs when there exists a point τ in {1, 2, …, n–1} such that the statistical properties of $\{y_1, y_2, \ldots, y_\tau\}$ and $\{y_{\tau+1}, y_{\tau+2}, \ldots, y_n\}$ are different in some characteristics. Regression CPs connect subsequent segments (i.e., adjacent linear segments) that differ from each other in levels of parameters of observed quantitative attributes. A model with the constraint that the linear segments are continuous at the CP (continuity constraint) is usually called a broken line regression model (*2*) or a joinpoint regression model (*3*).

To identify the optimal number of CPs (*3*), model selection is performed by using the grid-search method to fit the regression function with unknown joinpoints, assuming constant variance and uncorrelated errors. The number of significant joinpoints is found by performing a series of permutation tests; the procedure does not require asymptotic normality and maintains the correct Type I error probability level. The permutation procedure tests all possible locations of predetermined discrete CP locations in the data, specified by the settings, and identifies the optimum CP on the basis of the value of a cost function over all possible combinations. The Monte Carlo method is used to find the individual test's p value, and the Bonferroni correction is used to maintain the overall asymptotic significance level. These tests are also extended to the situation with nonconstant variance to handle rates with Poisson variation and possibly autocorrelated errors. Details are provided elsewhere (*3*,*4*).

We applied a linear model for the joinpoint analysis of the mean and median age of LD cases over the study period; for the LD age-standardized incidence analysis, we applied a log-linear model. A log transformation is used for LD rates because they arise from a Poisson distribution, which is skewed. Another reason is the ease of interpretation: under a log-linear model, the rates change at a constant percent per year (i.e., a fixed annual percent change), whereas for a linear model the rates change at a constant fixed amount per year. When comparing trends (e.g., across sites where the rates can be very different), the annual percent change is an appropriate metric for comparing across widely different scales.

For the mean and median age regression, a weighted least squares analysis was performed under the assumption of heteroscedastic (correlated) errors; standard errors for each study year were provided as input. In both models, we specified the minimum number of observations between 2 jointpoints to be 2 and the minimum number of observations from a jointpoint to either end of the data to be 4. The number of possible CPs was specified to be between 0 and 3.

References

1. National Cancer Institute. Joinpoint trend analysis software [cited 2021 October 25]. https://surveillance.cancer.gov/joinpoint

2. Siegmund DO, Zhang H. Confidence regions in broken line regression. Lect Notes Monogr Ser. 1994;23:292–316. https://doi.org/10.1214/lnms/1215463132

3. Kim HJ, Fay MP, Feuer EJ, Midthune DN. Permutation tests for joinpoint regression with applications to cancer rates. Stat Med. 2000;19:335–51. PubMed https://doi.org/10.1002/(SICI)1097-0258(20000215)19:3<335::AID-SIM336>3.0.CO;2-Z

4. Kim HJ, Yu B, Feuer EJ. Selecting the number of change-points in segmented line regression. Stat Sin. 2009;19:597–609. PubMed

Appendix Table. Age-standardized average incidence of Legionnaires' disease (cases/100,000 population), United States, 1992–2002 and 2003–2018

Demographic	1992–2002	2003–2018
Age group (not standardized), y		
0–4	0.03	0.03
5–14	0.02	0.01
15–24	0.07	0.10
25–34	0.18	0.37
35–44	0.38	0.90
45–54	0.66	1.90
55–64	1.02	3.09
65–74	1.42	3.83
75–84	1.57	4.50
≥85	1.49	5.52
Sex		
M	0.63	1.80
F	0.35	0.91
Race		
Native American/Alaska Native	0.26	0.48
Asian/Pacific Islander	0.14	0.36
Black/African American	0.47	2.15
White	0.37	0.99
Region/division/jurisdiction		
Northeast	0.68	2.34
New England	0.61	1.64
Connecticut	0.52*	1.65
Maine	0.29	0.87
Massachusetts	0.49	1.59
New Hampshire	0.45	1.69
Rhode Island	1.58	3.00
Vermont	1.44	1.50
Middle Atlantic	0.71	2.59
New Jersey	0.35	1.90
New York City	0.40	2.90
New York State	0.78	2.93
Pennsylvania	1.05	2.58
South	0.33	1.01
South Atlantic	0.44	1.17
Delaware	1.68	2.16
District of Columbia	1.56	3.81
Florida	0.33	1.01
Georgia	0.30	0.80
Maryland	0.92	2.41
North Carolina	0.27	0.98
South Carolina	0.35	0.57
Virginia	0.49	1.27
West Virginia	Not Reportable	1.58
East South Central	0.32	1.04
Alabama	0.15	0.67
Kentucky	0.44	1.42
Mississippi	0.22	0.57
Tennessee	0.42	1.28
West South Central	0.15	0.69
Arkansas	0.12	0.71
Louisiana	0.19	0.52
Oklahoma	0.28	0.68

Demographic	1992–2002	2003–2018
Texas	0.12	0.72
Midwest	0.67	1.67
East North Central	0.77	2.01
Illinois	0.31	1.51
Indiana	0.82	1.45
Michigan	0.75	2.10
Ohio	1.18	2.99
Wisconsin	0.91	1.53
West North Central	0.42	0.89
Iowa	0.54	0.65
Kansas	0.19	0.56
Minnesota	0.33	0.89
Missouri	0.45	1.20
Nebraska	0.83	0.81
North Dakota	0.26	0.52
South Dakota	0.42	1.01
West	0.29	0.66
Mountain	0.43	0.74
Arizona	0.38	0.76
Colorado	0.35	0.83
Idaho	0.25	0.58
Montana	0.48	0.56
Nevada	0.62	0.59
New Mexico	0.19	0.50
Utah	0.88	1.16
Wyoming	0.73	0.57
Pacific	0.23	0.63
Alaska	0.10	0.21
California	0.22	0.69
Hawaii	0.31	0.31
Oregon	Not Reportable	0.54
Washington	0.27	0.49
USA	0.48	1.33

*Not reportable in Connecticut 1992–1996.
